# Quality of Life in Patients with Chronic Low Back Pain and Differences by Sex: A Longitudinal Study

**DOI:** 10.3390/jpm14050496

**Published:** 2024-05-08

**Authors:** Xavier Pericot-Mozo, Rosa Suñer-Soler, Glòria Reig-Garcia, Josefina Patiño-Masó, Miquel Sitjar-Suñer, Afra Masià-Plana, Carme Bertran-Noguer

**Affiliations:** 1 Pain Unit, Institut Català de la Salut, 17007 Girona, Spain; xpericot@gmail.com; 2 Department of Nursing, Faculty of Nursing, University of Girona, 17004 Girona, Spain; gloria.reig@udg.edu (G.R.-G.); josefina.patino@udg.edu (J.P.-M.); miquel.sitjar@udg.edu (M.S.-S.); afra.masia@udg.edu (A.M.-P.); carme.bertran@udg.edu (C.B.-N.)

**Keywords:** chronic low back pain, health-related quality of life, functional limitation, nursing care

## Abstract

Background: The experience of chronic low back pain has a significant impact on the quality of life of affected people, resulting in difficulties in performing basic activities of daily living. Aim: To study the perceived quality of life of people affected by chronic low back pain and the associated factors by sex. Methods: A prospective, longitudinal and observational design was used. Results: A total of 129 people (58.1% women) with chronic low back pain were studied. The mean pain intensity scores were of moderate severity (6.42 points), with a modest improvement at follow-up (6.17 points). Epidural nerve blocks were the most effective therapeutic intervention in reducing the intensity of pain. Participants described a negative perception of their health with regard to quality of life, with low scores for the two constructs both at baseline (health index, 0.444; perception of health, 38.76 points) and at follow-up (health index, 0.447; perception of health, 40.43 points). Participants had severe functional limitation scores (50.79 points). The results were significantly better among men. There was an inverse relationship between the average pain intensity (β = −0.304; *p* < 0.001), functional limitation (β = −0.466; *p* < 0.001) and mental health (β = −0.565; *p* < 0.001) and quality of life. Conclusions: The chronification of low back pain complicates people’s biopsychosocial adaptation to life. There is a longitudinal inverse association between pain and functional limitation and health-related quality of life.

## 1. Introduction

Chronic low back pain (CLBP) is a persistent, complex musculoskeletal syndrome and is the most prevalent and disabling chronic disorder in the adult population worldwide [1], creating a heavy burden both socioeconomically [2] and in terms of healthcare assistance at health centres [3,4]. The aetiology is essentially chronic disc degeneration and inflammation in young people due to proinflammatory cytokines [5] and articular in the lumbar area in older people, which may be associated with intermittent neurogenic claudication in the legs [6]. 

It was found that it was not possible to determine the specific aetiology of CLBP in between 85% and 90% of people since the radiological evidence of the pathoanatomical lesion did not fully clarify the origin of the pain [7]. Therefore, in this respect, not only can the nociceptive and neuropathic perception of pain alter both the perception of health and the health of the patient, but other strongly related factors also have a significant influence. Biopsychosocial alterations, the social context, value systems, objectives, previous experiences and expectations can all modulate the experience of pain [8,9], and the impact will be reflected in people’s quality of life (QoL).

Chronic low back pain is particularly prevalent in older age groups and women [10,11] and is expected to increase in low- and middle-income countries [7]. A prevalence rate of 22.6% has been found in Catalonia [12], 13.69% in Spain [13] and between 12 and 30% in adults worldwide [1]. In people > 60 years old, the prevalence in several countries around the world oscillates between 50 and 60% [6,8]. 

Numerous studies have shown that psychological factors have a significant influence on the experience of pain and perceived health, determining the emotional response, coping style and recovery when faced with CLBP [14]. Positive cognitive variables (beliefs and emotions) and behavioural variables (acceptation and adaptation) mitigate the development, continuation and chronicity of CLBP and explain the individual differences with regard to tolerance, coping, perception and adaptation to pain by age and sex [15,16].

On the other hand, the constant presence of negative emotions [17], maladaptive coping strategies, catastrophism [18], somatisation, insomnia, depressive mood, widespread pain syndromes, central hypersensitivity syndromes and personality changes are significant predictive variables of worse adaptation to pain and worse perceived health and health [19] at all ages [20], but to a lesser extent in men [21].

There is currently a consensus that conservative non-pharmacological treatments, together with behavioural cognitive therapies [22,23,24,25,26,27], back school and physiotherapy [28,29,30,31], McKenzie therapy and Pilates [3] and mindfulness [32] are the most effective strategies to significantly improve pain, functional states, mental health, self-control and adaptive coping strategies, which is supported by strong evidence [33,34].

With regard to other variables, persistent daily functional limitation is the factor that most affects the QoL of people with CLBP in their activities of daily living, and this increases with age [35]. A lack of adequate knowledge regarding the quantity and type of exercises that can be performed was found. There is moderate evidence [3] that performing regular mild physical exercise with progressive moderate stretching [36] results in significant clinical differences in the reduction in perceived pain and functional limitation [3,37].

On the other hand, there is strong evidence that inactivity predisposes people to increased disability and mental disorders [29]. In this respect, daily functional limitation affects the usual occupational activity of workers. The associated disability, absence from work and invalidity create a greater risk of pain chronification, higher levels of pain and worse perceived health [33], which have high family and social costs [38], affecting men to a lesser extent [21]. The avoidance of certain occupational exposures, such as repetitive mechanical overloading, and the adoption of ergonomic habits at the workplace prevent the chronification of pain and the worsening of perceived pain and improve perceived health [8,37].

Maintaining good perceived family and social support [10], engaging in recreational and distraction activities and avoiding isolation are factors that indicate improvements in social functional limitation, adaptation and an improved prognosis for people with CLBP and help to avoid depressive moods [39]. Knowledge of these variables will allow us to improve the management and decision-making in coping with and adapting to CLBP.

The importance of the present study is in the fact that it strengthens the existing evidence regarding how chronic low back pain affects the quality of life of people who suffer from it, especially with regard to their mental health. Additionally, we study the relationship between the interventions undertaken by the Pain Unit and the evolution of pain.

The main aim was to study the quality of life of people affected by CLBP and the associated factors by sex. As well as describing the general characteristics of the participants and the aetiology and related risk factors, we investigated the intensity of the pain, perceived health and functional limitation. Our hypothesis was that CLBP affects the QoL of people who suffer from it, especially those people who report greater perceived pain and functional limitation.

## 2. Materials and Methods

### 2.1. Design

A longitudinal, observational and prospective design aimed to study people with CLBP, with a three-month follow-up, carried out at the Pain Unit of the Dr. Josep Trueta University Hospital.

### 2.2. Participants

The non-probabilistic convenience sample was composed of people making their first visit to the Pain Unit of the Dr. Josep Trueta University Hospital, diagnosed with CLBP, and who voluntarily agreed to participate in the study during the period running from December 2018 to July 2019.

The participants attended their first visits at the Pain Unit with a diagnosis of CLBP, referred from Traumatology or Neurosurgery, with MRI and/or electromyography diagnostic tests. The anaesthetists of the Pain Unit then performed their own clinical and physical examinations.

The inclusion criteria required participants to be adults ≥ 18 years old, living in the Girona Health Region and diagnosed with CLBP with or without radiculopathy, who, after reading the study information, agreed to participate in the study by signing the informed consent form. People who were under 18 years of age and those with cognitive deficiencies that impeded their ability to respond to the questionnaires were excluded from the study.

### 2.3. Data Collection

An ad hoc self-administered questionnaire was designed to record sociodemographic data (age, sex, living arrangements), risk factors (sedentarism, smoking status, alcohol use, occupational distress) and health-related variables (BMI, history of anxiety and depression, sleep alterations, history of surgical interventions, other chronic diseases, treatments with nerve blocks, pharmacological treatments).

#### 2.3.1. Pain Intensity

In order to study pain, the Spanish version of the Brief Pain Inventory Short Form (BPI-SF) of Cleeland and Ryan [40] by Badia et al. [41] was used, with a Cronbach’s alpha coefficient > 0.7. The BPI is a multidimensional instrument that evaluates pain characteristics and the location of the pain, with 4 items measuring the pain intensity (where 0 represents “No pain” and 10 “Pain as bad as you can imagine”) and 7 items measuring how the pain has interfered with the respondent’s activities of daily living (where 0 represents “Doesn’t interfere” and 10 “Completely interferes”). The questionnaire is composed of 15 items. All responses are given on a numerical scale from 0 to 10 (where 0 corresponds to not having pain and 10 to the worst pain imaginable).

#### 2.3.2. Health-Related Quality of Life

In order to measure perceived QoL with regard to the health of the study participants, the Spanish version of the EuroQol Quality of Life Scale (EQ-5D-3L) [42] by Badia et al. [43] was used, with a Cronbach’s alpha coefficient was 0.8. This scale has two components: health state description and evaluation. In the description part, patients are required to report on five dimensions of their health status, namely mobility (walking ability), self-care (the ability to wash or dress by oneself), usual activities (performance in work, study, housework, etc.), pain/discomfort (how much pain or discomfort they experience) and anxiety/depression (how anxious or depressed they are), on a scale of 1–3 (from no problems to extreme problems). Patients self-rate their level of severity for each dimension using a three-level scale. Health states can therefore be described using a five-digit number. Altogether, the descriptive system generates a total of 243 possible health states. Each health state is converted into a single EQ-5D-3L health rating that oscillates between 1 (no health problem), representing the best health state, and 0 (worst health state), which corresponds to death. The calculation of the score is performed based on the evaluation of the population in several different countries [44]. In the evaluation part, patients evaluate their overall health status using a visual analogue scale (VAS) from 0 to 100 (from 0 “The worst health you can imagine” to 100 “The best health you can imagine”) [45].

#### 2.3.3. Disability

In order to evaluate the level of disability and functional limitation in daily activities among people with CLBP, the Oswestry Disability Index (ODI) by Fairbank et al. [46], in its Spanish version devised by Flórez et al. [47], was used, where the Cronbach’s alpha coefficient was 0.85. The ODI consists of 10 items: pain intensity, personal care, lifting, walking, sitting, standing, sleeping, sex life, social life and travelling. Each item is scored on a scale of 0 to 5 points, where 0 represents “no limitation” and 5 the “maximum limitation”. The total score is obtained from the sum of the scores for each question, which is then divided by the number of responses obtained and multiplied by 100: 0–20% (minimal disability), 21–40% (moderate disability), 41–60% (severe disability), 61–80% (crippling back pain) and 81–100% (bedbound or with the exacerbation of symptoms). A higher score indicates greater functional limitation due to back problems.

### 2.4. Description of Procedure and Data Collection

The first collection of data, corresponding to the first phase of the study, was conducted by the researcher at the nursing clinic. The researcher gave information about the study to those people who attended the Pain Unit on their first visit and who met the inclusion criteria. Participants were given the study information sheet and signed an informed consent form.

The second collection of data, corresponding to the second phase of the study, was conducted by the same researcher at the same nursing clinic three months after the date of the first collection of data. The same participants again filled in the same self-administered questionnaires as before.

### 2.5. Ethical Considerations

This study respected the current ethical norms for studies on humans. The project was presented to the Ethics and Clinical Research Committee of the reference area before the start of the research study for its evaluation and received its approval (QdVDLC-2018122).

### 2.6. Statistical Analysis

The statistical study was performed using the IBM SPSS 27 software. Continuous variables are described as the mean and standard deviation or the median and interquartile range. Categorical variables are described by the absolute frequency and their percentages. The chi-squared test and/or the Fisher test were used to study associations between categorical variables. Quantitative variables were compared using Student’s *t*-test. In addition, a logistic regression model was applied to explain the relationship between the perceived quality of life of participants and the associated factors by sex. In all tests, significance was taken as *p* < 0.05 with a 95% confidence interval.

## 3. Results

One hundred and twenty-nine people were included, with an average age of 62.5 years (SD = 15.29). The ages of the participants were between 21 and 89 years and 58.1% were women. Overall, 72.1% lived in families and 41.1% were retired. Moreover, 41.9% had a lack of personal autonomy, requiring the assistance of other people in their basic daily life activities, which was more common in women.

CLBP was mechanical and degenerative, with radicular pain in the legs (93.8%), in all age groups, and this was greater in women. Surgical interventions were lumbar discectomy (52.4%) and lumbar arthrodesis (47.6%). Overall, 89.1% followed a pharmacological treatment, which was more common in women. The main analgesics used were opioids (49.7%), followed by paracetamol (36.4%) and gabapentinoids (34.1%).

In total, 73.6% of the participants stated that they had experienced mental health disorders, such as anxiety (93.8%), depression (87.6%) and sleep alterations (58.9%). Sedentarism (91.1%), a lack of leisure activities (60%) and being overweight or obese (BMI of 29.49 Kg/m^2^), which was significantly less common among women, were observed to be modifiable risks. Occupational risks were intense, such as constant physical overload with poor posture (58.9%), which was more common in men, and occupational stress (31%), which was higher in women.

### 3.1. Intensity of Perceived Pain in Activities of Daily Living in People with CLBP at Follow-Up

The scores for the intensity of pain and its interference with activities of daily living were slightly better at follow-up. Specifically, statistically significant differences were observed in the maximum intensity of pain, the mean intensity of pain, mood, the individual occupation, relationships with family and friends and sleep. Women obtained significantly worse scores in all items. Furthermore, men referred to a greater perception of pain relief (Table 1).

### 3.2. Perceived Health of People with CLBP Related to QoL at Follow-Up

Participants gave unfavourable average scores for perceived health at follow-up, which had a major impact on QoL and the general health state. Low scores were observed both in the health index and in perceived general health. The dimensions that most affected QoL were pain/discomfort and mental disorders (anxiety and depression), with statistically significantly better scores in men (Table 2). Women also had worse total scores for the health index and the health state (Table 3).

### 3.3. Functional Limitation of People with CLBP Related to QoL at Follow-Up

A high percentage of functional limitation was observed at the follow-up (50.79%). The dimensions related to activities of daily living with the greatest limitation were sexual activity, pain intensity, standing up, social life and lifting weights. The highest percentages obtained in the responses to the questionnaire were “My sex life is nearly absent because of pain” with 93%, “The pain is very severe at the moment” with 70%, “Pain prevents me from standing for more than 10 min” with 68% and “Pain has restricted my social life to my home” with 56%, without differences between the sexes.

### 3.4. Relationships among Variables Associated with QoL

Perceived health correlated positively and significantly with the health state and negatively with the pain intensity and physical function limitation. Age correlated positively and significantly with the pain intensity and functional limitation.

In the multiple linear regression model analysing the QoL of the participants at follow-up, a significant inverse relationship was found between the pain intensity, psychological disorders and functional limitation. In other words, the greater the pain intensity, the lower the perceived health, and the lower the functional limitation, the greater the perceived health (Table 4).

## 4. Discussion

This study has investigated the perceived health, health state and functional limitation of 129 people affected by CLBP and the associated factors by sex with a three-month follow-up.

### 4.1. General Characteristics of Participants with CLBP

Different sociodemographic factors have been observed to have an influence on QoL. With regard to age, in general, a higher mean age was found than in other studies that we have consulted. Our result was the same as in Rutledge et al. [48], but the mean age was lower in Gouteron et al. [49], Fullen et al. [50] and Boekel et al. [51]. Few studies have evaluated CLBP in people > 60 years old. The same results were found in the systematic review and meta-analysis of Wong et al. [6]; older age was significantly associated with a greater perception of pain and functional limitation, and with worse perceived health and states of physical health in general, worse QoL and a greater risk of CLBP. In our study, as in most studies, a low educational level was found in many participants (75.1%), which was even lower than in Mutubuki et al. [38].

With regard to sex, there were slightly more women than men (58.1%), which was similar to the vast majority of studies, including Boekel et al. [51] with 57%, Jegan et al. [52] with 57.7% and Tyack et al. [53] with 58.4%. According to the systematic review of Meucci et al. [54], these sex-based differences could be related to the vulnerability of women, due to the greater burden of tasks that they take on in the home, their employment in different occupational activities, their biological characteristics, pregnancy and post-menopausal hormonal processes.

There are several modifiable risk factors that are involved in the reduction of QoL. A high level of sedentarism was observed in both sexes in the present study (91.5%), as in Coluccia et al. [55] and Hong and Shin [56]. Being overweight was significantly more common in men (IMC of 29.49 kg/m^2^), a result that was the same as in Quentin et al. [36] and Ruiz et al. [57], and similar to Mutubuki et al. [38]. Furthermore, more than half of the participants showed a lack of adaptative strategies in leisure and distraction. CLBP sufferers did not participate in any leisure or sporting activity in their free time, nor did they engage in conservative multidisciplinary activities. The affected people were more vulnerable since those who wished to perform activities could not do so due to the persistent functional limitation as a result of either the intensity of the pain or the avoidance of movement in order to avoid pain, which was more common in men.

In the present study, CLBP was found to have a major mental health impact, as in other studies [21]. Seven out of ten people referred to having mental health disorders, which was significantly higher in women, with results that were similar to those in Valdés et al. [58] (76.73%). The main disorders observed during follow-up were anxiety (reactivity and attention), depression (isolation) and sleep alterations [5], with results that were similar to those of Hong and Shin [56] and Ünal et al. [59]. These disorders are associated with greater comorbidity [55], chronicity and greater negative emotional responses [60].

Moreover, CLBP is found to have a continued significant impact on productivity at work. Although four out of ten people in the present study were retired, among those who were still working, we found that they had great difficulty in performing their professional duties normally, with occupational distress, absences from work and long and frequent temporary periods of occupational incapacity, with men being less affected [3]. All of these factors have a significant influence on perceived QoL [10].

### 4.2. Intensity of Lumbar Pain

The perception of pain was of moderately high intensity, which was similar to Ghai et al. [61] and Ramírez et al. [62]. Women reported a significantly greater perception of pain and more interference in all basic aspects of their daily lives, as in Agnus et al. [10] and Zavarize and Wechsler [21]. As an aggravating factor, it was observed that 93.8% of people reported radiculopathy in the legs, which was slightly higher in women.

The most effective therapeutic intervention observed was the use of epidural steroid injections (level 1 evidence), performed during the period of the study, with an improvement of two points in the maximum pain intensity and in the mean pain intensity, as in Manchikanti et al. [63]. It should be noted that in the systematic review of Cho et al. [64], it was pointed out that epidural blocks proved not to be as effective in people with failed back surgery. With regard to pain relief obtained as a result of pharmacological treatment, a certain improvement was found at follow-up, with men reporting greater relief. Other authors highlight that rehabilitation treatments and educational interventions can reduce perceived pain by more than five points (from 7.17 to 2.78 points on the VAS) [14] and improve the functional limitation [65].

### 4.3. Perceived QoL

The QoL related to health is probably the most widely used indicator for the evaluation of the subjective perception of health, the health state and wellbeing with regard to chronic diseases. The results obtained in this study are coherent with the other studies that we have consulted. Most participants continue to manifest negative perceived health and health states in their experiences with CLBP, and this has a great impact on their activities of daily living.

In this respect, very low scores were observed for the health index (EQ-5D-3L), both at baseline (value of 0.444) and follow-up (0.447). These results were similar to those of Boekel et al. [51], who found a value of 0.39; Ghai et al. [61], with a value of 0.35; Mutubuki et al. [38], with a value of 0.48; Ramírez et al. [62], with a value of 0.451; and Ruiz et al. [57], with a value of 0.451. With regard to the general health state, low scores were observed both at baseline (38.76 points) and follow-up (40.43). Men had significantly higher scores in both cases.

During the follow-up of the study, it was also observed that persistent pain produced significant multidimensional problems and a reduction in QoL related to health, with some differences by sex. These were especially high and significant in the dimensions of pain (57.4%) and mental health disorders (70.5%), with alterations in all age groups, and this was even more accentuated in women [15]. There were certain problems in the dimensions of mobility, daily activities and self-care, with men being more strongly affected. Similar results were found in Obradovic et al. [66]. Other studies using a different QoL questionnaire, such as the SF-36, including Ünal et al. [59], have also obtained similar results in all age groups.

### 4.4. Functional Limitation

CLBP causes a restriction in people’s physical functional capacity and worse adaptation in the basic and necessary activities of daily living in normal conditions. This chronic functional deterioration causes incapacity and has a large negative impact on people’s perception of pain and perceived health, and on their health.

Physical functional limitation was one of the most important factors affecting the participants, with more problems and major limitations, in the present study. Some authors have highlighted that CLBP results in more years lived with functional incapacity than any other health condition worldwide [67]. It was observed globally that all people studied during the follow-up presented percentages of functional limitation of intense severity that allowed them to perform the basic activities of daily living with normality, as in Ghai et al. [61] (51%). Higher percentages were found in those of an older age, as in Jegan et al. [52] (53.7%) and Ungureanu et al. [68] (56.19%), and lower percentages with a younger age, as in Van Dongen et al. [69] (42.7%), Garcia et al. [70] (39.6%) and Ünal et al. [59] (18.4%). One possible explanation for this is that, in these latter studies, the mean age of the participants was younger, and these patients typically undertake more preventative therapeutic exercises than older people [3]. In the present study, the age was higher, and so there was natural lumbar articular degeneration. Wong et al. [6] concluded that physical inactivity could explain this functional limitation and stressed the importance of following a programme of physical activity. In this respect, a global postural re-education program is effective compared to other exercise programmes in subjects with persistent chronic low back pain [71].

In our study, although the participants had been able to adapt to a certain extent to some daily functional limitations, we highlight, by order of effect, sexual incapacity (72.1%), difficulties in undertaking journeys (56.6%), drugs not alleviating the pain (54.3%), difficulties in standing (51.9%), difficulties in sitting, being unable to sleep and difficulties in picking up objects, walking and personal care.

The results of the present study confirm the hypothesis that CLBP negatively affects the QoL of people who suffer from it, and especially those who refer to having a greater intensity of pain and greater physical function limitation. Similar results have been described in the studies of Agnus et al. [10], Járomi et al. [8], Palit et al. [72] and Zavarize and Wechsler [21].

This is one of the few longitudinal studies to analyse the relationship between the characteristics of patients with CLBP, the aetiology of pain, risk factors, clinical variables in relation to the intensity of pain and physical function limitation.

The results suggest to us that patients should be incorporated as active agents in the healthcare system, ensuring that they are ready to cooperate and share in taking responsibility for their health, strengthening and improving protective factors and adopting prevention strategies to improve their daily lives. This change in mentality, both in people in general and in healthcare professionals in the paradigm of care for people with CLBP, could be considered as a new line of investigation to advance in the process of empowerment and the acceptance of CLBP and of people’s adaptation to it.

### 4.5. Limitations

Although the study was longitudinal, a longer study period would have made it possible to detect variations in pain crises. Most reviews and meta-analytical studies have highlighted the significant heterogeneity in the methodological criteria of the studies considered. A methodological focus aimed at reducing the great heterogeneity in the definition of concepts is essential allow a comprehensive understanding and comparative analysis of different studies.

Furthermore, it should be noted in interpreting the main results of this study that the chronification of lumbar pain may have led to the values observed for the pain intensity not being as high as they were in the acute phase, and that the participants could have become accustomed to certain limitations in their daily functional activity.

With regard to the blocks that were used, it should be noted that the waiting time to receive a block in the Pain Unit was very long, with the result that we were only able to evaluate pain relief as a result of this intervention in a limited number of people in our study period.

## 5. Conclusions

The results of this study show that the participants had negative perceived health and health statuses in their experience of chronic lumbar pain and that this had a significant impact on their daily lives, with high levels of pain intensity and functional limitation being observed, especially in women. However, perceived health correlates positively and significantly with pain intensity and physical function limitations. There is a significant inverse relationship between pain intensity, psychological disorders and functional limitation and perceived health.

Strengths: The longitudinal design made it possible to follow the evolution of chronic low back pain and its association with the evolution of mental health and quality of life.

Weaknesses: A longer period of study might have led to the detection of further possible variabilities. Furthermore, few patients received nerve block treatment, limiting our ability to conduct a conclusive analysis of its efficacy.

## Figures and Tables

**Table 1:** The intensity of the pain and the effects on the basic aspects of daily life at baseline and at follow-up.

Brief Pain Inventory	Baseline	Follow-Up	*p*
Your pain at its worst in the last 24 h	7.76 (2.10)	7.47 (2.08)	0.015
Your pain at its least in the last 24 h	5.08 (2.68)	4.79 (2.66)	0.108
Your pain on average	6.42 (2.24)	6.17 (2.21)	0.055
How much pain you have right now	6.21 (2.72)	5.98 (2.37)	0.177
How much relief have pain treatments or medications provided in the last 24 h (%)	23.22%	30.12%	
Pain has interfered with your general activity during the past 24 h	6.95 (2.04)	6.80 (2.07)	0.200
Pain has interfered with your mood during the past 24 h	7.91 (3.10)	7.27 (3.28)	<0.001
Pain has interfered with your walking ability during the past 24 h	6.47 (3.32)	6.41 (3.18)	0.286
Pain has interfered with your normal work during the past 24 h	6.82 (2.45)	6.50 (2.41)	0.023
Pain has interfered with your relationship with other people during the past 24 h	5.85 (2.54)	5.50 (2.42)	0.018
Pain has interfered with your sleep during the past 24 h	4.57 (4.02)	4.19 (3.80)	0.026
Pain has interfered with your enjoyment of life during the past 24 h	8.44 (2.05)	8.26 (2.09)	0.134

The results are expressed as the mean and standard deviation (SD).

**Table 2:** The dimensions of the EQ-5D-3L for chronic low back pain at baseline and follow-up for the overall sample and by sex.

EQ-5D-3L	Baseline			*p*	During Follow-Up			*p*
	Sample	Men	Women		Sample	Men	Women	
	N: 129	N: 54 (41.9%)	N: 75 (58.1%)		N: 129	N: 54 (41.9%)	N: 75 (58.1%)	
**Mobility**								
I have no problems in walking about	74 (57.4)	34 (63)	40 (53.3)		72 (55.8)	34 (63)	38 (50.7)	0.209
I have some problems in walking about	55 (42.6)	20 (37)	35 (46.7)	0.286	57 (44.2)	20 (37)	37 (49.3)	
I am confined to bed	0 (0)	0 (0)	0 (0)		0 (0)	0 (0)	0 (0)	
**Self-care**								
I have no problems with self-care	118 (91.4)	50 (92.6)	68 (90.7)		117 (90.7)	51 (94.4)	66 (88)	0.401
I have some problems washing/dressing myself	10 (7.8)	4 (7.4)	6 (8)	0.688	11 (8.5)	3 (5.6)	8 (10.7)	
I am unable to wash or dress myself	1 (0.8)	0 (0)	1 (1.3)		1 (0.8)	0 (0)	1 (1.3)	
**Usual activities**								
I have no problems with my usual activities	105 (81.4)	46 (85.2)	59 (78.7)		104 (80.6)	47 (87)	57 (76)	0.249
I have some problems with my usual activities	23 (17.8)	8 (14.8)	15 (20)	0.507	24 (18.6)	7 (13)	17 (22.7)	
I have unable to perform my usual activities	1 (0.8)	0 (0)	1 (1.3)		1 (0.8)	0 (0)	1 (1.3)	
**Pain/Discomfort**								
I have no pain or discomfort	2 (1.6)	1 (1.9)	1 (1.3)		2 (1.6)	1 (1.9)	1 (1.3)	0.014
I have moderate pain or discomfort	53 (41)	28 (51.8)	25 (33.3)	0.097	55 (42.6)	31 (57.4)	24 (32)	
I have extreme pain or discomfort	74 (57.4)	25 (46.3)	49 (65.4)		72 (55.8)	22 (40.7)	50 (66.7)	
**Anxiety/Depression**								
I am not anxious or depressed	10 (7.8)	5 (9.3)	5 (6.7)		11 (8.5)	6 (11.1)	5 (6.7)	0.033
I am moderately anxious or depressed	28 (21.7)	17 (31.4)	11 (14.6)	0.049	28 (21.7)	17 (31.5)	11 (14.6)	
I am extremely anxious or depressed	91 (70.5)	32 (59.3)	59 (78.7)		90 (69.8)	31 (57.4)	59 (78.7)	

The results are expressed as the mean and standard deviation (SD) or median and interquartile range [IQR].

**Table 3:** The health index and health state for chronic low back pain at baseline and follow-up for the overall sample and by sex.

	Baseline				During Follow-Up			
	Sample	Men	Women	*p*	Sample	Men	Women	*p*
	N: 129	N: 54 (41.9%)	N: 75 (58.1%)		N: 129	N: 54 (41.9%)	N: 75 (58.1%)	
**Health index**	0.444 (0.16) 0.416 [0.327–0.476]	0.481 (0.16) 0.457 [0.384–0.493]	0.417 (0.16) 0.416 [0.327–0.476]	0.030	0.447 (0.17) 0.416 [0.327–0.476]	0.500 (0.17) 0.468 [0.384–0.739]	0.408 (0.15) 0.416 [0.327–0.476]	0.002
**Health state**	38.76 (27.47) 40 [20–60]	44.72 (27.2) 50 [25–65]	34.47 (27.04) 40 [10–50]	0.036	40.43 (27.3) 45 [20–60]	47.31 (27.65) 50 [20–70]	35.47 (26.12) 40 [10–50]	0.014

The results are expressed as the mean and standard deviation (SD) or median and interquartile range [IQR].

**Table 4:** The linear regression model of QoL *perceived* (N: 129).

	Unstandardised Coefficient		Standardised Coefficient	*t*	*p*	95% Confidence Interval for B	
	B	ES	β			Lower Limit	Upper Limit
(Constant)	1.058	0.049		21.480	<0.001	0.960	1.155
Age	6.271	0.001	0.006	0.074	0.941	−0.002	0.002
Sex	−0.052	0.027	−0.150	−1.968	0.051	−0.105	0.000
Average pain intensity	−0.024	0.006	−0.304	−3.680	<0.001	−0.036	0.011
Functional limitation	−0.005	0.001	−0.466	−8.414	<0.001	−0.006	−0.004
Mental disorders	−0.151	0.014	−0.565	−10.653	<0.001	−0.179	−0.123

B: unstandardised coefficient; ES: standard deviation error; β: standardised β coefficient; A: 0.828; R^2^: 0.686; adjusted R^2^: 0.679.

## Data Availability

The data supporting the findings of this study are available upon request from the corresponding author.
